# Impact of a Multimodal Intervention Combining Manual Therapy, Exercise, Reduced Methylxanthine Intake, and Nocturnal Light Avoidance on Inflammatory and Metabolic Profiles, Pain, Functionality, and Sleep Quality in Patients with Frozen Shoulder: A Single-Blind Randomized Controlled Trial

**DOI:** 10.3390/jcm14134539

**Published:** 2025-06-26

**Authors:** Rafael Guzmán-García, María Pérez-Montalbán, Leo Pruimboom, Santiago Navarro-Ledesma

**Affiliations:** 1 Medicine and Public Health PhD Program, Faculty of Health Sciences, University of Granada, Av. de la Ilustración, 60, 18071 Granada, Spain; rguzmangarcia@correo.ugr.es; 2 Department of Physiotherapy, Faculty of Nursing, Physiotherapy and Podiatry, University of Sevilla, C. Avenzoar, 6, 41009 Sevilla, Spain; mapermont@gmail.com; 3 Department of Physiotherapy, University Chair in Clinical Psychoneuroimmunology (University of Granada and PNI Europe), 2518 JP The Hague, The Netherlands; leo@cpnieurope.com; 4 Department of Physiotherapy, Faculty of Health Sciences, Campus of Melilla, University of Granada, Querol Street 5, 52004 Melilla, Spain

**Keywords:** circadian, frozen shoulder, inflammation, metabolism, sleep

## Abstract

**Background:** Frozen shoulder (FS) is a common musculoskeletal condition with significant socioeconomic impact. Despite its prevalence, the condition lacks a definitive understanding and universally effective treatment approach. **Objective:** To evaluate the effects of an intervention combining manual therapy, conventional exercises, and strategies to improve sleep quality and circadian rhythm on recovery and biomarkers in patients with FS. **Methods:** A single-blind, randomized, controlled trial was conducted with 34 participants divided into control and experimental groups (n = 17 each). Both groups received manual therapy and conventional exercises, while the experimental group (EG) also received sleep and circadian rhythm optimization instructions. Biomarkers (fasting glucose, insulin, Homeostasis Model Assessment of Insulin Resistance (HOMA) index, leptin, triglycerides, total cholesterol, HDL cholesterol, uric acid, CRP, IL-1β, IL-6, IL-17, IL-10, IL-33, HMGB1, and TNF-α) and functional outcomes (SPADI, ROM, and PSQI) were assessed pre- and post-intervention. **Results:** After six weeks, the EG showed significant improvements in IL-10 levels (mean change: 2.5 pg/mL vs. 0.5 pg/mL in the control group (CG), *p* = 0.03), IL-6 reduction (−1.8 pg/mL vs. −0.4 pg/mL, *p* = 0.02), and HOMA index (−0.8 vs. −0.2, *p* = 0.04). ROM improved by 20 degrees in the EG versus 10 degrees in the CG (*p* = 0.01), SPADI scores decreased by 25 points versus 15 points (*p* = 0.03), and PSQI improved by 4 points compared to 2 points (*p* = 0.05). **Conclusion:** The integration of sleep quality and circadian rhythm optimization into conventional rehabilitation significantly enhances recovery, particularly IL-10 modulation, but these did not translate into superior clinical improvements within the study period. Further long-term studies are needed to confirm whether early biological effects lead to sustained functional recovery in FS patients.

## 1. Introduction

Frozen shoulder (FS) is a complex musculoskeletal condition characterized by the spontaneous fibrosis of the glenohumeral joint capsule, associated with a complex immune-mediated inflammatory response [1]. This condition primarily manifests as persistent pain and marked limitation of both active and passive shoulder joint mobility [2,3]. The symptoms that FS patients experience negatively impact daily activities, work capacity, and quality of life [4,5]. Epidemiologically, FS has a prevalence ranging from 2% to 5% of the general population, with a notably higher incidence in women aged 40–60 years [6,7]. It is worth noting that the frequency of this condition increases with age, suggesting a possible relationship with degenerative and metabolic changes associated with aging [8].

Frozen shoulder has historically been referred to using multiple terms, including “adhesive capsulitis.” However, the recent literature highlights that this term may not accurately reflect the underlying pathology, as adhesions are not consistently present and the inflammatory phase is typically transient. Current recommendations from international societies, such as ISAKOS and ASES, advise against using “adhesive capsulitis”, as the term “frozen shoulder” is widely accepted by both clinicians and patients and better represents the clinical presentation of the condition [9].

The etiology of FS is multifactorial and not yet fully understood [10]. Recent research has highlighted the importance of psycho-emotional factors in its development and progression [11,12]. At the cellular and molecular levels, detailed studies have revealed the presence of a complex inflammatory infiltrate in affected tissues, including immune cells, inflammatory mediators [13], and elevated matrix metalloproteinase (MMP) activity [14]. Recent investigations have identified an inflammatory profile in the joint capsule of FS patients, characterized by the dysregulation of key cytokines such as interleukin 1 beta (IL-1β), interleukin 6 (IL-6), interleukin 17 (IL-17), interleukin 10 (IL-10), interleukin 33 (IL-33), high mobility group B1 protein (HMGB1), C-reactive protein (CRP), and tumor necrosis factor alpha (TNF-α) [15]. These pro-inflammatory molecules play a crucial role in fibroblast activation and dysregulated collagen synthesis, contributing to the fibrosis characteristic of this condition. In this vein, a recent meta-analysis demonstrated the influence of both metabolic and inflammatory blood profiles in patients with frozen shoulder FS. HbA1c and cholesterol emerged as the most strongly associated metabolic biomarkers, while IL-1β and TNF-α were closely linked to inflammation and fibrosis. These findings underscore the need for further research evaluating changes in metabolic and inflammatory markers following therapeutic interventions [16]. However, it shares overlapping features with other neuromusculoskeletal conditions that must be considered during differential diagnosis, particularly in early stages. One such condition is Parsonage–Turner Syndrome (PTS)—also known as neuralgic amyotrophy—a rare brachial plexopathy that typically presents with sudden-onset severe shoulder pain followed by muscle weakness and, occasionally, scapular winging or sensory disturbances. Although less common than FS, PTS can mimic the early pain-dominant phase of FS, leading to potential diagnostic confusion. Distinguishing features include a more abrupt onset, the presence of neurological deficits (e.g., deltoid or biceps weakness), and electromyographic findings consistent with brachial plexus involvement, which are not typical of FS. Including a careful neuromuscular examination and, where needed, referral for electrodiagnostic studies can help confirm FS diagnosis and avoid misclassification. Given its clinical overlap, the exclusion of PTS and other mimicking conditions is essential for appropriate patient selection in interventional studies targeting frozen shoulder [17].

Another factor directly impacting general health is sleep. Sleep plays a fundamental role in regulating numerous physiological functions, including inflammatory response and tissue repair. Chronic sleep disruption or deprivation has been associated with increased circulating levels of multiple proinflammatory substance, potentially exacerbating and perpetuating the inflammatory state in FS [18,19]. Specifically, in the context of this condition, the persistent pain experienced by many patients, even at rest, can significantly disrupt their circadian rhythm and sleep architecture, and vice versa [20,21,22]. Two external factors have demonstrated significant impact on sleep quality and, by extension, on the regulation of inflammatory processes: methylxanthine intake and blue light exposure. Methylxanthines, a class of compounds including caffeine, theobromine, and theophylline, are commonly found in everyday foods and beverages such as coffee, tea, and chocolate. These compounds have been shown to alter various sleep parameters, primarily through their antagonistic action on adenosine receptors [23,24,25]. Additionally, nocturnal exposure to artificial light, especially light emitted by electronic devices, can adversely affect the circadian rhythm. Mammals, including humans, are particularly susceptible to circadian disruptions caused by even low levels of artificial light exposure at night [26,27,28,29].

Research on FS has targeted developing and evaluating effective interventions to improve mobility and reduce pain. In this regard, standard rehabilitation approaches typically include manual therapy and exercise interventions [30,31]. Clinical guidelines recommend individualized programs combining joint mobilization, capsular stretching, patient education, and exercise progression based on the patient’s irritability level and disease stage [32]. However, despite the demonstrated importance of sleep quality on health, no studies have specifically target interventions aimed at improving it and there is a need for research in this area [33]. Therefore, the aim of this study was to evaluate the effects of a multimodal intervention combining manual therapy, conventional exercise, and chronobiological strategies—specifically the reduction of methylxanthine intake and the avoidance of nocturnal light exposure—on systemic blood markers related to inflammation and metabolism, as well as on sleep quality, pain levels, and shoulder function in patients with frozen shoulder. The present study hypothesizes that the group receiving the combined intervention of manual therapy, exercise, reduced methylxanthine intake, and nocturnal light avoidance would show greater improvements in inflammatory and metabolic profiles, sleep quality, pain reduction, and functional recovery compared to the group receiving manual therapy and exercise alone.

## 2. Materials and Methods

### 2.1. Design

A randomized, controlled, single-blind clinical trial was conducted. The study was registered on 2 May 2024 in the Protocols and Results Registry System (PRS) of ClinicalTrials.gov (NCT06409871), adhered to the CONSORT recommendations for clinical trials [34], and was approved by the Ethics Committee of the Reina Sofia Hospital in Córdoba (Spain) and the Research Ethics Committees of Andalusia, Spain (SICEIA-2024-001305). The trial commenced shortly after registration.

### 2.2. Patient Cohort

Patients were recruited from a private clinic (Clínica Metódica) in Cordoba, Spain. A total of 34 individuals diagnosed with primary FS, aged between 18 to 60 years, were included. Symptom duration at inclusion ranged between three months and two years, corresponding primarily to the freezing or frozen stages of FS capsulitis as described in current clinical guidelines [32]. Exclusion criteria were previous occurrences of locked dislocations, arthritis, or shoulder fractures; avascular necrosis; prior surgeries in the hypochondriac region within the past year; or any medical or skin conditions preventing tactile stimuli in the shoulder region. Neurological or motor disorders, diagnosed psychopathologies, visual disabilities, or other diseases affecting sleep quality or inflammatory parameters were also exclusion criteria. Prior to the study’s initiation, all participants were fully informed of the general aspects of the study and provided written informed consent.

### 2.3. Procedures

**Figure 1:**
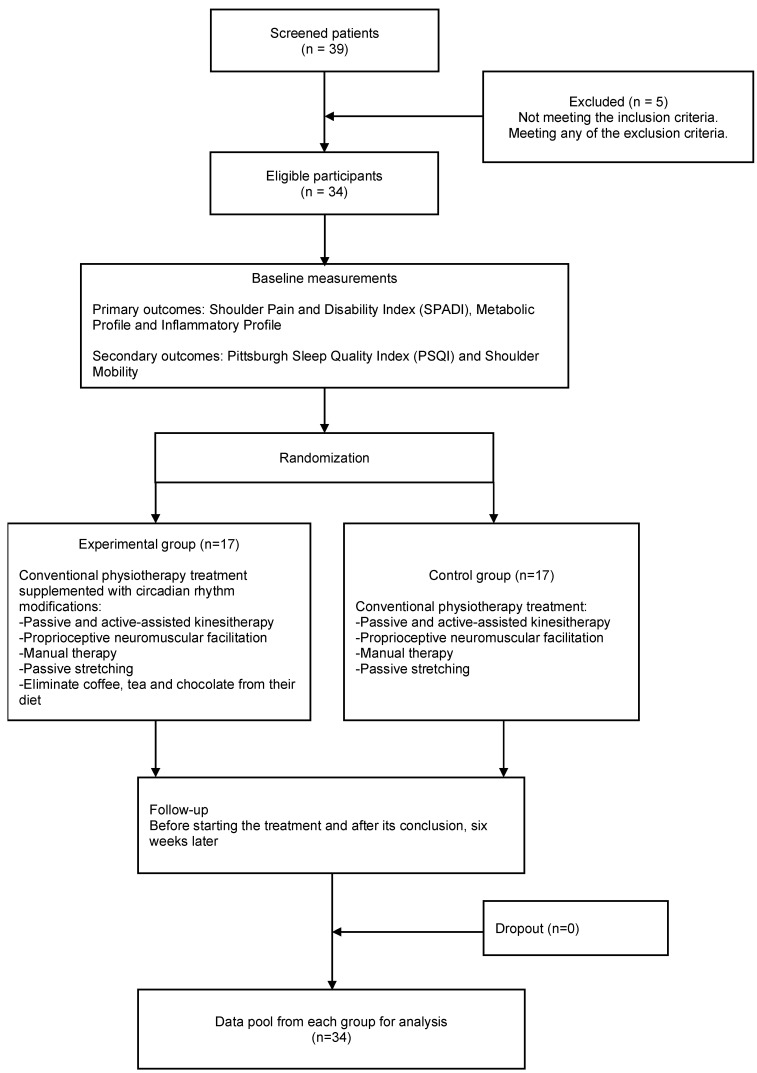
Flow diagram.

A total of 39 participants were screened, with 5 being excluded: 2 due to oncological conditions and 3 due to psychiatric medication use that could significantly affect sleep, making them ineligible for the study. Participants were randomly assigned to one of two groups: the control group (CG, n = 17) or the experimental group (EG, n = 17). Randomization was performed using Excel software, with the sequence maintained by an independent collaborator to ensure allocation concealment (see Figure 1). This study was conducted as a single-blind, randomized, controlled trial. Outcome assessors and physiotherapists responsible for evaluations were blinded to group allocation to minimize detection and performance bias. However, due to the nature of the experimental intervention—which included dietary modifications (elimination of methylxanthines) and behavioral recommendations (avoidance of blue light exposure before bedtime)—participant blinding was not feasible, and the individuals in the experimental group may have been aware of their allocation.

Patients in the CG received conventional physiotherapy treatment based on current clinical guidelines for FS. This included the following:

Passive and active-assisted kinesitherapy to address flexion/extension and abduction/adduction movements of the shoulder, along with internal/external tilting, scapular elevation and depression, and scapular abduction and adduction [35,36]; proprioceptive neuromuscular facilitation using Kabat’s diagonals [37]; manual therapy, including deep transverse massage (Cyriax technique) on the cervical spine, scapula, and rotator cuff [36]; and passive stretching of the trapezius, latissimus dorsi, subscapularis, external rotators, and pectoralis muscles [35]. Symptom irritability was clinically considered during each treatment session to guide the progression of interventions, in line with current guidelines [38]. Detailed information on physiotherapy treatment can be found in the Supplementary Materials.

Patients in the EG received the same physiotherapy treatment, supplemented with circadian rhythm modifications. This intervention included eliminating methylxanthine-rich foods and beverages (coffee, tea, and chocolate) from their diet and avoiding exposure to electronic devices for two hours before bedtime [39,40,41,42,43,44].

Participants documented these modifications in a diary over six weeks, noting bedtime, wake-up time, and adherence to circadian rhythm adjustments. A second informed consent form was provided to this group, including a data collection commitment. The six-week intervention period was selected based on previous research demonstrating that structured interventions can lead to measurable improvements in sleep quality and inflammatory response within similar timeframes [45,46].

Both groups received only the assigned treatment for FS. The interventions consisted of 50 min sessions, twice weekly, for a total of 12 sessions. A minimum of 48 h was required between sessions. The same physiotherapist administered the interventions to both groups while remaining blinded to the rest of the processes.

### 2.4. Evaluations

All measurements were recorded before starting the treatment and after its conclusion six weeks later. These evaluations were performed by the same evaluator, a physiotherapist different from the one applying the interventions, who remained blinded to the group assignments. Data were stored in an Excel document.

#### 2.4.1. Primary Outcomes

Shoulder Pain and Disability Index (SPADI): A quality-of-life questionnaire developed to evaluate pain and disability associated with shoulder dysfunction. The SPADI consists of 13 items and is widely used for shoulder function assessment [47,48]. Test/retest reliability of the SPADI total and subscale scores ranged from 0.6377 to 0.6552. Internal consistency ranged from 0.8604 to 0.9507. SPADI total and subscale scores were highly negatively correlated with shoulder range of motion (ROM) supporting the criterion validity of the index [47,48].

Metabolic Profile: Fasting glucose, insulin, Homeostasis Model Assessment of Insulin Resistance (HOMA) index (it is used to assess insulin sensitivity, calculated from fasting glucose and insulin levels), leptin, triglycerides, total cholesterol, HDL cholesterol, uric acid, and high-sensitivity C-reactive protein (CRP). Blood samples were obtained following the guidelines of the National Biobank Network [49]. The blood samples were collected at baseline and post-intervention and plasma samples were stored at -80°C until analysis.

For fasting glucose, triglycerides, total cholesterol, HDL cholesterol, uric acid, and CPR, sample tubes were centrifuged with inert separator gel, provided by the Nursing Department, at a speed of 3000 rpm for 10 min. After centrifugation, 400 μL of the serum obtained was pipetted into a pediatric well compatible with the A15 analyzer from Biosystems. This device automatically performs the determinations using turbidimetry, employing specific reagents for each measurement. For LDL cholesterol determination, LabData was used, which is a LIMS (Laboratory Information Management System). This system automatically applies Friedewald’s formula:

Cholesterol LDL = Total Cholesterol − Cholesterol HDL − (Triglycerides/5)

The HOMA index was calculated based on fasting glucose and insulin values, as defined by the formula

HOMA-IR = {Glucose [mg/dL] × 0.0555 × Insulin [UI/mL]} × 22.5

Inflammatory Profile: Inflammatory cytokines, including IL-1, IL-6, IL-17, IL-10, IL-33, HMGB1, CRP, and TNF. The analysis of these inflammatory cytokines was conducted using ELISA (Enzyme-Linked Immunosorbent Assay), with blood samples obtained according to the guidelines of the National Biobank Network [49]. The Invitrogen ELISA kit was used with pre-coated wells, meaning that antibodies were already adhered to the base of the wells. First, the included wash buffer was diluted with deionized water following the protocol described in the specific kit. The protein standard was reconstituted and allowed to stand for approximately 10 min. A serial dilution of the standard was performed by first adding a small amount of diluent to each well in the strip included in the kit and then pipetting the reconstituted protein into the first well according to the described protocol. Generally, this is accomplished by mixing and pipetting 250 μL from one well to another until reaching the seventh well. Standards, controls, and samples are then added to the ELISA plate included in the kit. Finish by diluting the concentrated assay buffer with distilled water.

All extractions were performed following a homogeneous protocol. Specifically, all blood samples were collected between 8:45 and 10:00 am to reduce circadian variability in the parameters analyzed. Participants attended after a minimum of 8 h of fasting, as explicitly indicated to them the day before collection by a telephone call confirming the appointment. All collections were performed by the same nurse in the collection room of the day hospital, thus ensuring consistency in the procedure and minimizing possible biases associated with interprofessional or environmental variability. All participants had their blood drawn with the forearm supported and in extension on a stand, following the recommended standard position, which contributed to patient comfort and homogeneity of the procedure.

#### 2.4.2. Secondary Outcomes

Pittsburgh Sleep Quality Index (PSQI): A questionnaire that evaluates sleep quality and disturbances. It consists of 19 questions covering a wide range of factors related to sleep quality, including estimates of sleep duration, latency, and the frequency and severity of sleep-related problems [50,51]. Acceptable measures of internal homogeneity, consistency (test/retest reliability), and validity have been shown. A global PSQI score greater than 5 has shown a diagnostic sensitivity of 89.6% and specificity of 86.5% (kappa = 0.75, p less than 0.001) in distinguishing good and poor sleepers [50,51].

Shoulder Mobility: Shoulder range of motion was assessed using an inclinometer and following the established protocols [52].

#### 2.4.3. Sample Size

Based on previous studies that applied physiotherapy interventions in individuals with FS (with a mean SPADI score of 66 points and a standard deviation (SD) of 16) and considering the Minimal Detectable Change (MDC) of 17 points reported by Tveita et al. [53], a sample size was calculated using Jamovi software (Version 2.3.26) to detect a 16-point difference between groups, with 80% power and a significance level of 0.05. The required total sample size was 30 patients (15 per group). Accounting for a 15% dropout rate, the sample size was increased to 34 patients (17 per group).

### 2.5. Statistical Analyses

Statistical analyses were performed using SPSS software [version 23.0; SPSS Inc., Chicago, IL, USA)], and data are presented as mean ± standard deviation for normally distributed data or median with interquartile range for non-normally distributed data. Statistical analyses were conducted to evaluate both inter-group and intra-group differences pre- and post-intervention. Continuous variables were first assessed for normality using the Shapiro–Wilk test. For variables demonstrating normal distribution, parametric tests were employed, while non-normally distributed variables were analyzed with non-parametric tests. To determine intra-group pre- and post-intervention changes within each group, paired *t*-tests were used for normally distributed variables, whereas the Wilcoxon signed-rank test was applied to variables that did not meet normality assumptions. The significance level for intra-group comparisons was set at *p* < 0.05, and effect sizes were calculated to interpret the magnitude of the intervention within each group. Inter-group comparisons were analyzed using independent *t*-tests for normally distributed variables, and the Mann–Whitney U test was employed for non-normally distributed variables. To address potential Type I errors due to multiple comparisons, the Bonferroni correction was applied, adjusting the significance threshold accordingly. Cohen’s d was calculated for parametric tests to determine the magnitude of differences between groups, with thresholds defined as small (0.2), medium (0.5), and large (0.8). For non-parametric tests, the rank-biserial correlation was used to evaluate effect sizes, following the same magnitude thresholds.

## 3. Results

A total of 34 subjects participated in the study (see Figure 1). No significant differences were observed in baseline characteristics between the experimental and control groups, ensuring comparability prior to the intervention (see Table 1).

### 3.1. Intra-Group Changes

The experimental group showed significant improvements (*p* < 0.05) in most biomarkers and outcome measures after the intervention: insulin, HOMA-IR, IL-1, IL-6, IL-10, TNF-α, leptin, glucose, HDL cholesterol, and C-reactive protein (CRP). Furthermore, functional and quality-of-life measures (Pittsburgh Sleep Quality Index, Shoulder Pain and Disability Index (SPADI), and range of motion) showed improvements after the intervention (see Table 2 and Table 3). The control group showed significant improvement only in CRP. Functional improvements were shown in SPADI and ROM. All data related to the statistical analyses are available in Table 2, Table 3 and Table 4.

A total of 34 participants were included in the study. Blood samples were collected at two time points: pre-intervention (baseline) and post-intervention (after six weeks). Data are presented as mean ± standard deviation (SD) for variables with normal distribution and as median [interquartile range (IQR)] for non-normally distributed variables, as determined by the Shapiro–Wilk test. † shows all non-normally distributed variables.

Several biomarkers and range of motion (ROM) variables (flexion, extension, adduction, internal and external rotation) were non-normally distributed and analyzed using non-parametric tests. Specifically, within-group comparisons (pre/post) were performed using the paired *t*-test for normally distributed variables and the Wilcoxon signed-rank test for non-normally distributed variables. Between-group comparisons were conducted using the independent *t*-test for parametric variables and the Mann–Whitney U test for non-parametric variables.

### 3.2. Inter-Group Comparisons (n = 17 per Group)

The only biomarker showing a statistically significant difference between groups post-intervention was IL-10 (*p* < 0.05). No significant inter-group differences were observed in other biomarkers or functional measures, although trends favoring the experimental group were noted in sleep quality and inflammatory markers (see Table 4).

## 4. Discussion

This study aimed to evaluate the impact of an intervention combining manual therapy, conventional exercises, and strategies to improve sleep quality and circadian rhythm on patients with FS, specifically eliminating methylxanthine intake (coffee, tea, and chocolate) and avoiding exposure to blue-light-emitting devices before bedtime. The findings revealed significant intra-group improvements in most biomarkers and functional outcomes in the experimental group, particularly in the anti-inflammatory cytokine IL-10, compared to the control group. However, no between-group differences were found in functional outcomes. These results suggest that enhancing sleep and circadian rhythm may play a pivotal role in modulating inflammation and promoting recovery in this patient population.

Our findings highlight the importance of incorporating sleep and circadian rhythm optimization into conventional rehabilitation protocols for FS [33,54]. The experimental intervention led to significant improvements in key biomarkers, particularly IL-10, which exhibited a statistically significant difference between groups [55,56]. While IL-10 is known for its anti-inflammatory properties and its role in resolving inflammation by inhibiting pro-inflammatory cytokines and promoting macrophage polarization towards an M2 phenotype, the observed increase in IL-10 should be interpreted with caution, likely as an association rather than direct causality [57,58]. Although both groups demonstrated improvement in functional outcomes (SPADI and ROM), the absence of statistically significant between-group differences in these clinical variables prevents us from directly linking IL-10 changes to functional improvements. While both groups showed improvements in pain and functional outcomes measured by the SPADI and ROM, no statistically significant differences were observed between them. This indicates that, although the experimental intervention led to favorable biological changes—particularly an increase in IL-10—these did not result in greater functional recovery within the 6-week study period. It is important to interpret these findings cautiously, as functional improvements in frozen shoulder typically occur progressively over time. Clinical recovery may lag behind initial changes in inflammatory profiles. Previous longitudinal studies have shown that ROM and shoulder disability scores improve gradually, and that factors such as baseline ROM, diabetes, and central pain processing can influence clinical trajectories [59,60,61]. Therefore, while the current findings support a potential immunomodulatory benefit of the intervention, longer follow-up is needed to determine whether these early biological responses are associated with clinically meaningful gains in pain and function. Further research is required to determine whether this modulation of IL-10 contributes directly to joint capsule recovery or fibrosis resolution in FS patients. The results suggest a potential mechanism by which improved sleep quality and circadian rhythm may enhance recovery processes [62,63]. Moreover, while the observed IL-10 increase in the experimental group aligns with its known role in inflammation resolution and tissue remodeling, its biological significance in vivo remains to be fully elucidated in frozen shoulder [56]. Current evidence suggests that IL-10 modulates macrophage polarization, fibrosis reduction, and joint recovery; however, whether the IL-10 changes observed in this study correlate with meaningful inflammation reduction or functional improvements still requires further investigation [64,65,66]. Future studies should assess whether IL-10 enhancement through sleep and circadian interventions directly impacts macrophage phenotypic shifts, extracellular matrix remodeling, and joint capsule fibrosis resolution in FS patients, providing a clearer mechanistic understanding of its therapeutic relevance.

Previous studies have demonstrated the link between sleep disturbances and increased inflammation, with reduced IL-10 levels being a common finding in individuals with poor sleep [67,68,69]. The observed increase in IL-10 in the experimental group may reflect the synergy between improved circadian regulation and immune modulation [62,70]. This aligns with emerging evidence that circadian rhythm influences the timing and magnitude of cytokine release, including IL-10, highlighting its potential as a therapeutic target for inflammatory conditions [62,70,71]. Nevertheless, while functional improvements were observed in both groups, the absence of inter-group differences in SPADI and ROM measures suggests that these clinical outcomes cannot be attributed solely to changes in IL-10. The functional improvements observed in SPADI and ROM also underscore the broader clinical impact of the intervention. While these measures improved in both groups, the more extensive changes in the experimental group suggest that targeting systemic inflammation through non-pharmacological means, such as optimizing sleep, can accelerate recovery.

The relationship between IL-10 and tissue remodeling in chronic inflammation must be further detailed. In the context of FS, a condition characterized by inflammation and fibrosis of the joint capsule, IL-10 may play a crucial role in mediating tissue remodeling and resolution of inflammation, as seen in previous studies [72,73]. This cytokine exerts its effects by suppressing the production of pro-inflammatory mediators, such as TNF-α, IL-6, and IL-1β, and promoting macrophage polarization toward the M2 phenotype [74,75]. The M2 macrophages are associated with anti-inflammatory and tissue-repair functions, which are critical in preventing chronic fibrosis and promoting recovery in conditions like frozen shoulder [76]. IL-10 also regulates extracellular matrix (ECM) remodeling by modulating the activity of matrix metalloproteinases (MMPs) and inhibiting excessive fibrotic deposition. This dual role—suppressing inflammation and facilitating ECM remodeling—is particularly relevant in FS, where prolonged inflammation can lead to joint stiffness and fibrosis. While the intervention may have influenced this process through IL-10 elevation, this remains speculative and requires verification in future studies [76].

In addition, the role of circadian rhythm regulation in macrophage polarization should be highlighted, since they are critical in regulating macrophage function, including their polarization into pro-inflammatory (M1) or anti-inflammatory (M2) phenotypes. This polarization is central to resolving inflammation and promoting tissue repair, processes essential for conditions like FS. The circadian clock controls the timing of cytokine release and macrophage activity through molecular feedback loops involving core clock genes such as BMAL1, CLOCK, PER, and CRY [77]. Macrophages in the M2 phenotype, which are driven by IL-10, demonstrate enhanced anti-inflammatory and tissue-repair functions. Disruption of circadian rhythms has been shown to skew macrophage activity toward an M1 phenotype, leading to prolonged inflammation. For instance, Curtis et al. noted that deletion of BMAL1 in macrophages leads to increased pro-inflammatory cytokine production, such as IL-6 and TNF-α, disrupting the balance required for inflammation resolution [77]. In addition, the influence of circadian clocks extends to immunometabolism, where BMAL1 suppresses glycolysis—a process linked to M1 polarization—while promoting oxidative phosphorylation, which supports M2 polarization. This metabolic regulation underpins the immune response’s timing and efficacy in resolving inflammation [77]. In our study, the intervention targeting sleep and circadian rhythm may have enhanced the circadian regulation of macrophage polarization, increasing IL-10 production and promoting the anti-inflammatory M2 phenotype. This mechanism aligns with the observed improvements in functional outcomes (e.g., ROM and SPADI scores) and highlights the broader impact of circadian alignment on recovery processes in frozen shoulder [78,79]. Although our intervention was designed to support circadian rhythm alignment, no circadian markers were measured in the present study, and conclusions regarding this pathway remain hypothetical.

Furthermore, improved sleep quality is intricately linked to the regulation of inflammatory responses, particularly through cytokines like IL-10. Sleep deprivation has been shown to exacerbate systemic inflammation, leading to increased levels of pro-inflammatory cytokines such as IL-6 and TNF-α while reducing IL-10, an essential anti-inflammatory mediator. Although IL-6 levels did not show statistically significant differences between groups, a notable intra-group reduction was observed in the experimental group. This finding may indicate a trend towards reduced systemic inflammation following the multimodal intervention. IL-6 is a key pro-inflammatory cytokine involved in both acute and chronic inflammatory responses, and its reduction has been associated with improved clinical outcomes in musculoskeletal disorders. The observed decrease, despite not reaching inter-group significance, aligns with previous evidence linking sleep optimization and circadian rhythm regulation to downregulation of IL-6 expression [69,80]. It is plausible that the improved sleep quality and reduced nocturnal light exposure contributed to a favorable immunomodulatory effect, warranting further investigation in larger, longer-term studies. The aforementioned imbalance (increased levels of pro-inflammatory cytokines such as IL-6 and TNF-α and decreased IL-10) can prolong the inflammatory state, impair tissue repair, and contribute to the persistence of chronic conditions [65]. The multifaceted nature of IL-10, as highlighted by Carlini et al., includes its role in suppressing harmful immune responses and promoting tissue homeostasis [65]. IL-10 achieves this through the Jak1/Tyk2 and STAT3 signaling pathways, which inhibit pro-inflammatory cytokine production and enhance tissue repair processes. Sleep optimization, as a modulator of IL-10 expression, could play a crucial role in mitigating inflammation and supporting recovery in chronic conditions like FS [65,79,81,82,83].

Methylxanthines are alkaloids that can interfere with the development and progression of frozen shoulder by generating significant biochemical effects on dopaminergic pathways, pain and inflammation modulation, sleep regulation, and chronobiology in general, among others. These alkaloids are found in higher concentrations in coffee (caffeine), chocolate (theobromine), and tea (theophylline) [84]. The mechanisms of action of methylxanthines are diverse, but the one with the greatest impact on frozen shoulder is their ability to act as non-selective antagonists on adenosine A1 and A2a receptors, which is why prolonged consumption leads to dependence. They are central nervous system stimulants, increase motor activity and intellectual performance, and decrease fatigue and sleepiness. These pharmacological effects can trigger a cascade of deleterious reactions affecting the recovery of frozen shoulder.

Although it is true that the metabolism of methylxanthines is strongly influenced by the individual’s genotype and phenotype, the half-life of these alkaloids is 3–5 h in normal metabolizers of these substances. If there is a polymorphism in CYP1A2, the elimination rate is considerably higher, so the consumption of coffee mainly, chocolate, and/or tea can lead to hypertension, insomnia, altered glucose levels, etc. [85,86]. Considering that more than 33% of the European population has the CYP1A2*1F (-163C>A) polymorphism, which is associated with lower enzymatic activity and therefore slower elimination of methylxanthines, we consider it necessary to make this chronobiological regulation intervention to assess the impact that the consumption of these substances may have on the progression of frozen shoulder. Exposure to blue light emitted by electronic devices such as mobile phones, tablets, televisions, computers, etc., at night can negatively interfere with the recovery of frozen shoulder. Blue light (especially wavelengths close to 469 nanometers) suppresses the release of melatonin, the sleep-inducing hormone. At night, this suppression delays the onset of sleep and reduces its quality, which is critical for muscle repair, reduction in inflammation, and cell regeneration, circumstances that slow recovery and can increase stiffness and pain perception in frozen shoulder [87,88,89,90].

### 4.1. Strengths and Weaknesses

This study has several notable strengths. It introduces a novel intervention combining sleep and circadian rhythm optimization, specifically, the reduction of methylxanthine intake and avoidance of nocturnal blue-light exposure, with conventional rehabilitation techniques, offering a holistic approach to managing FS. The inclusion of a robust biomarker analysis, particularly focusing on IL-10, provides mechanistic insights into the physiological pathways underlying recovery. Additionally, the use of validated functional outcome measures, such as SPADI and ROM, ensures clinically relevant evaluations of patient progress. The randomized, controlled trial design further enhances the reliability of the findings by minimizing bias and improving internal validity.

However, several limitations must be acknowledged. The relatively small sample size may limit the generalizability of the findings and reduce the power to detect between-group differences in several variables. The short duration of the intervention and follow-up period (6 weeks) may have been insufficient to observe functional improvements that typically manifest over longer recovery trajectories in FS. Although both groups improved in SPADI and ROM scores, no statistically significant differences were observed between them, suggesting that the biological changes detected—particularly in IL-10—may precede or act independently from clinical recovery in the short term. The lack of longer follow-up restricts our ability to determine whether the observed changes in inflammatory markers ultimately translate into sustained functional benefits. Although outcome assessors were blinded to group allocation, the nature of the intervention prevented participant blinding, introducing a potential source of performance and expectancy bias that should be considered when interpreting the results. The physiotherapy protocol used in this study was determined by the standard practices of the participating rehabilitation center. While it followed principles aligned with clinical guidelines—such as respecting tissue irritability—it did not include certain evidence-supported techniques [91]. This may limit the generalizability of our results to settings using more contemporary treatment approaches. Furthermore, the study did not assess muscle strength, an important determinant of functional capacity in FS patients and a variable that could offer additional context to the observed improvements. While IL-10 was emphasized as a key biomarker, the study did not fully explore its interactions with other inflammatory mediators such as IL-6 or TNF-α. In addition, clinical staging of frozen shoulder was not formally performed, and irritability levels were not documented at baseline, which limits interpretation of potential differences in disease progression. These factors should be addressed in future trials.

Finally, sleep quality was assessed through self-reported questionnaires, which, although validated, may introduce recall bias. The use of wearable technologies, such as actigraphy or circadian phase tracking devices, could have provided more objective and granular data regarding sleep patterns and circadian alignment. Incorporating such tools would enhance the accuracy of sleep assessments and improve the understanding of how chronobiological interventions influence systemic inflammation and patient function over time.

### 4.2. Future Research

Future studies should aim to address the limitations of the current research and explore new directions to build on its findings. Longitudinal studies with extended follow-up periods are needed to evaluate the sustained effects of sleep and circadian rhythm optimization on chronic inflammation and functional recovery. Larger, multicenter trials involving diverse populations would enhance the generalizability and external validity of the findings. Mechanistic investigations using advanced techniques, such as transcriptomics or metabolomics, could provide deeper insights into the molecular pathways through which IL-10 and circadian regulation influence tissue repair and inflammation resolution.

A potential avenue for future research would be to conduct a more in-depth analysis of the broader metabolic and inflammatory biomarker changes observed within the experimental group. While our study primarily focuses on biomarkers showing significant inter-group differences, the widespread intra-group improvements suggest that sleep and circadian rhythm optimization may have a more comprehensive impact on systemic health. Longitudinal studies with extended follow-ups and mechanistic analyses could provide further insight into the long-term effects of these interventions on metabolic regulation and inflammation resolution. Also, in the future, it should be considered that many markers do not follow a normal distribution, and this may alter the interrelation of results.

Given that functional outcomes did not differ significantly between groups, future studies should explore whether longer-duration interventions or follow-up periods could reveal delayed clinical benefits associated with early immunomodulatory changes. The use of strength measurements and functional capacity assessments would help determine whether biomarker modulation translates into meaningful musculoskeletal recovery.

Research should also focus on exploring the interplay between IL-10 and other cytokines, such as IL-6 and TNF-α, and metabolic factors to develop a more holistic understanding of the immune response in FS [92]. Stratifying participants by frozen shoulder stage or irritability levels at baseline—factors known to influence prognosis—could help refine clinical interpretations and reveal subgroup-specific effects of the intervention. Investigating personalized sleep interventions tailored to individual circadian chronotypes could optimize therapeutic outcomes.

Furthermore, expanding the intervention to other chronic inflammatory conditions, such as rheumatoid arthritis or tendinopathy, could validate its broader applicability and therapeutic potential. Finally, an area that remains unexplored is the potential relationship between FS and circadian misalignment in shift or night workers. Given the well-established link between disrupted circadian rhythms, chronic inflammation, and impaired tissue repair, future studies should investigate whether individuals with irregular sleep patterns or shift work schedules exhibit a higher prevalence or altered progression of FS. Understanding this association could provide further insights into the role of circadian regulation in musculoskeletal health and support the development of targeted interventions for at-risk populations.

## 5. Conclusions

This study aimed to evaluate the effects of a combined intervention of manual therapy, exercise, reduced methylxanthine intake, and nocturnal light on systemic inflammatory and metabolic markers, sleep quality, pain, and shoulder function in patients with FS. The findings revealed a statistically significant increase in IL-10 levels in the experimental group compared to controls, alongside improvements in inflammatory and metabolic markers. However, no significant between-group differences were observed in clinical outcomes such as range of motion or SPADI scores. These results suggest that targeting inflammation through non-pharmacological strategies may modulate biological recovery pathways, but further longitudinal studies are needed to determine whether these changes translate into sustained functional benefits over time.

## Figures and Tables

**Table 1:** Sociodemographic and clinical characteristics of the participants.

	Experimental Group (n = 17)	Control Group (n = 17)	Shapiro–Wilk
	Count/Mean-SD	Count/Mean-SD	*p*
Age	50.2–10.2	52.1–12.5	0.373
Weight (kg)	69.4–10.8	67.8–12.0	0.308
Height (cm)	171–4.69	169–6.11	0.612
BMI	23.8–3.00	23.9–4.10	0.093
Sex			
Female	14	11	
Male	3	6	
Comorbidities			
Diabetes type 2	4	2	
Hypertension	5	0	
Chondromalacia patellae	3	0	
Hypothyroidism	1	3	
Obstructive sleep apnea	0	2	

Note. SD, standard deviation; BMI, body mass index; kg, kilograms; cm, centimeters.

**Table 2:** Descriptive table.

	Experimental Group (n = 17)	Control Group (n = 17)	
	Mean/Media–SD/IQR	Mean/Median–SD/IQR	*p*
Basal insulin (mU/L)	18.00-10.90	12.00–11.90	0.026 *–0.163 †
HOMA	4.840–3.440	2.370–2.470	0.002 *–0.148 †
IL-1 (pg/mL)	8.300–3.600	5.300–2.150	0.001 *–0.076 †
IL-6 (pg/mL)	4.070–3.740	1.960–1.830	<0.001 *–0.048 †
IL-10 (pg/mL)	1.170–1.030	1.140–0.700	<0.001 *–0.469 †
IL-17 (pg/mL)	1.210–1.030	1.200–0.440	<0.001 *–0.654 †
IL-33 (pg/mL)	123.0–124.0	23.40–114.0	<0.001 *–0.278 †
TNF (pg/mL)	15.43–4.795	14.14–3.777	0.353 *–0.392
HMBG1 (ug/L)	1.100–0.850	0.830–0.780	0.016 *–0.158 †
Leptin (ng/mL)	8.300–8.070	6.300–6.000	<0.001 *–0.134 †
Glucose (mg/dL)	108.82–16.353	100.0–23.06	0.107 *–0.207
Triglycerides (mg/dL)	132.35–50.241	122.7–48.11	0.081 *–0.574
Total cholesterol (mg/dL)	231.2–31.40	233.4–29.00	0.028 *–0.428 †
HDL cholesterol (mg/dL)	51.76–12.770	57.84–15.40	0.139 *–0.219
LDL cholesterol (mg/dL)	156.59–26.686	155.8–27.68	0.310 *–0.939
Uric acid (mg/dL)	4.400–2.700	4.300–1.700	0.035 *–0.904 †
C-reactive protein (mg/L)	5.500–2.800	4.100–2.680	<0.001 *–0.148 †
Total PITT	7.000–6.00	7.000–4.00	0.021 *–0.808 †
SPADI total pain	48.50–8.590	47.76–8.930	0.050 *–0.816
SPADI total disc	68.10–11.70	64.70–13.50	0.526 *–0.445
ROM FLEX	130–35.0	100–60.0	0.024 *–0.214 †
ROM EXT	40.0–15.0	40.0–20.0	0.010*–0.333†
ROM ABD	122–34.2	100–30.1	0.105*–0.061
ROM ADD	20.0–5.00	20.0–15.0	0.001 *–0.703 †
ROM EXT ROT	50.0–25.0	50.0–15.0	0.004 *–0.889 †
ROM INTER ROT	50.0–15.0	50.0–15.0	0.005 *–0.661 †

Note. SD, standard deviation; IQR, interquartile range; mU/L, milliunits per liter; HOMA, HOMA index; IL, interleukin; pg/mL, picograms per milliliter; TNF, tumor necrosis factor; HMBG1, high mobility group box 1 protein; ug/L, micrograms per liter; ng/mL, nanograms per milliliter; mg/dL, milligrams of sugar per deciliter; mg/L, milligrams per liter; SPADI, Shoulder Pain and Disability Index; ROM, range of motion; FLEX, flexion; EXT, extension; ABD, abduction; ADD, adduction; EXT ROT, external rotation; INTER ROT, internal rotation. The *p*-value indicated by * is that determined by the Shapiro–Wilk test. All non-normally distributed variables are indicated in the tables with a cross symbol. † shows non-normally distributed variables.

**Table 3:** Within-group comparisons before and after the intervention.

	Experimental Group (n = 17)				Control Group (n = 17)			
	*p*	Mean Differences	95% CI	Cohen’s D Test	*p*	Mean Differences	95% CI	Cohen’s D Test
Basal insulin (mU/L)	<0.001	2.9471	1.5139/4.380	1.0572	0.105	2.4394	−0.5698/5.449	0.4168
HOMA	<0.001	1.1459	0.5808/1.711	1.0427	0.149	0.8171	−0.3252/1.959	0.3678
IL-1 (pg/mL)	0.003	2.4700	0.9599/3.980	0.8410	0.126	0.7224	−0.2261/1.671	0.3916
IL-6 (pg/mL)	<0.001	1.5735	0.8628/2.284	1.1383	0.078	0.5688	−0.0713/1.209	0.4569
IL-10 (pg/mL)	0.117	−0.5053	−1.1526/0.142	−0.4013	0.740	−0.0406	−0.2950/0.214	−0.0820
IL-17 (pg/mL)	0.224	0.1682	−0.1139/0.450	0.3066	0.217	0.1300	−0.0846/0.345	0.3114
IL-33 (pg/mL)	0.080	14.4794	−1.9350/30.894	0.4535	0.198	13.9294	−8.0787/35.938	0.3254
TNF (pg/mL)	0.003	2.4700	0.9526/3.987	0.8369	0.174	1.1647	−0.5687/2.898	0.3455
HMBG1 (ug/L)	0.105	0.1735	−0.0403/0.387	0.4173	0.340	0.1400	−0.1615/0.442	0.2387
Leptin (ng/mL)	<0.001	2.4894	1.4746/3.504	1.2612	0.273	1.1194	−0.9707/3.210	0.2754
Glucose (mg/dL)	0.009	6.2941	1.8201/10.768	0.7233	0.884	0.4706	−6.2310/7.172	0.0361
Triglycerides (mg/dL)	0.256	4.6471	−3.7147/13.009	0.2857	0.955	0.2941	−10.6908/11.279	0.0138
Total cholesterol (mg/dL)	0.597	1.7176	−5.0352/8.470	0.1308	0.217	5.4306	−3.5289/14.390	0.3116
HDL cholesterol (mg/dL)	0.007	−3.8588	−6.5237/−1.194	−0.7445	0.869	0.3353	−3.8949/4.565	0.0408
LDL cholesterol (mg/dL)	0.144	4.2335	−1.6016/10.069	0.3730	0.276	4.7353	−4.1745/13.645	0.2733
Uric acid (mg/dL)	0.954	0.0153	−0.5390/0.570	0.0142	0.906	0.0388	−0.6487/0.726	0.0290
C-reactive protein (mg/L)	<0.001	2.0118	0.9554/3.068	0.9792	0.023	1.3335	0.2068/2.460	0.6085
Total PITT	0.006	1.7647	0.5933/2.936	0.7745	0.332	−0.1765	−0.5506/0.198	−0.2425
SPADI total pain	<0.001	8.5294	6.9023/10.156	2.6953	<0.001	5.7059	4.1876/7.224	1.9322
SPADI total disc	<0.001	16.5294	14.0561/19.003	3.4362	<0.001	13.2941	10.8910/15.697	2.8443
ROM FLEX	<0.001	−12.3529	−15.2440/−9.462	−2.1969	<0.001	−4.4118	−6.4201/−2.403	−1.1295
ROM EXT	<0.001	−6.7647	−9.7711/−3.758	−1.1569	0.005	−3.8235	−6.3175/−1.330	−0.7882
ROM ABD	0.505	−4.4118	−18.1323/9.309	−0.1653	<0.001	−5.0000	−6.5743/−3.426	−1.6330
ROM ADD	<0.001	−4.1176	−5.9882/−2.247	−1.1318	<0.001	−4.4118	−6.2026/−2.621	−1.2666
ROM EXT ROT	<0.001	−10.2941	−13.7516/−6.837	−1.5308	0.020	−7.9412	−14.4358/−1.447	−0.6287
ROM INTER ROT	<0.001	−9.4118	−12.9181/−5.905	−1.3801	<0.001	−4.4118	−6.4201/−2.403	−1.1295

Note. CI, confidence interval; mU/L, milliunits per liter; HOMA, HOMA index; IL, interleukin; pg/mL, picograms per milliliter; TNF, tumor necrosis factor; HMBG1, high mobility group box 1 protein; ug/L, micrograms per liter; ng/mL, nanograms per milliliter; mg/dL, milligrams of sugar per deciliter; mg/L, milligrams per liter; SPADI, Shoulder Pain and Disability Index; ROM, range of motion; FLEX, flexion; EXT, extension; ABD, abduction; ADD, adduction; EXT ROT, external rotation; INTER ROT, internal rotation.

**Table 4:** Inter-group comparisons after the intervention.

	*p*	Mean Differences	Cohen’s D Test
Basal insulin (mU/L)	0.174 †	−2.71588	−0.39709
HOMA	0.099 †	−0.61588	−0.27536
IL-1 (pg/mL)	0.163 †	−0.65824	−0.20035
IL-6 (pg/mL)	0.058 †	−0.70706	−0.39293
IL-10 (pg/mL)	<0.001 †	−0.88353	−1.36375
IL-17 (pg/mL)	0.796 †	0.05941	0.07385
IL-33 (pg/mL)	0.168 †	−33.48706	−0.61773
TNF (pg/mL)	0.985	0.02118	0.00664
HMBG1 (ug/L)	0.262 †	−0.11765	−0.27389
Leptin (ng/mL)	0.196 †	−0.93000	−0.11987
Glucose (mg/dL)	0.550	−3.00000	−0.20717
Triglycerides (mg/dL)	0.741	−5.23529	−0.11432
Total cholesterol (mg/dL)	0.931 †	−0.74824	−0.02893
HDL cholesterol (mg/dL)	0.645	1.88824	0.15954
LDL cholesterol (mg/dL)	0.881	−1.21941	−0.05179
Uric acid (mg/dL)	0.945 †	−0.10941	−0.09972
C-reactive protein (mg/L)	0.945 †	−0.00294	−0.00137
Total PITT	0.376 †	1.52941	0.40731
SPADI total pain	0.518	2.11765	0.22432
SPADI total disc	0.980	−0.11765	−0.00868
ROM FLEX	0.060 †	−22.64706	−0.68478
ROM EXT	0.114 †	−7.35294	−0.54853
ROM ABD	0.063	−20.88235	−0.66191
ROM ADD	0.883 †	−0.29412	−0.05092
ROM EXT ROT	0.414 †	−2.64706	−0.23825
ROM INTER ROT	0.462 †	−3.52941	−0.31838

Note. mU/L, milliunits per liter; HOMA, HOMA index; IL, interleukin; pg/mL, picograms per milliliter; TNF, tumor necrosis factor; HMBG1, high mobility group box 1 protein; ug/L, micrograms per liter; ng/mL, nanograms per milliliter; mg/dL, milligrams of sugar per deciliter; mg/L, milligrams per liter; SPADI, Shoulder Pain and Disability Index; ROM, range of motion; FLEX, flexion; EXT, extension; ABD, abduction; ADD, adduction; EXT ROT, external rotation; INTER ROT, internal rotation. All non-normally distributed variables are indicated in the tables with a cross symbol.

## Data Availability

All data generated or analyzed during this study are included in this published article. Data sets generated and/or analyzed during this study are available from the corresponding author upon reasonable request. They are not publicly available due to compliance with ethical criteria.

## Supplementary Materials

The following supporting information can be downloaded at: https://www.mdpi.com/article/10.3390/jcm14134539/s1.
