# Supplementary material for: Impact of a Multimodal Intervention Combining Manual Therapy, Exercise, Reduced Methylxanthine Intake, and Nocturnal Light Avoidance on Inflammatory and Metabolic Profiles, Pain, Functionality, and Sleep Quality in Patients with Frozen Shoulder: A Single-Blind Randomized Controlled Trial
Source: J Clin Med. 2025 Jun 26;14(13):4539. doi: 10.3390/jcm14134539 (PMC12249947; doi:10.3390/jcm14134539)
Supplement: Supplementary file 1 [file jcm-14-04539-s001.zip › jcm-3701084-supplementary.pdf]

CONVENTIONAL PHYSIOTHERAPY TREATMENT

Passive and active-assisted kinesitherapy

Movements	Series with each shoulder	Repetitions with each shoulder	Duration in seconds
**Flexion-extension**	2	10	3-5
**Abduction-adduction**	2	10	3-5
**Internal-external tilting**	2	10	3-5
**Scapular elevation-depression**	2	10	3-5
**Scapular abduction-adduction**	2	10	3-5

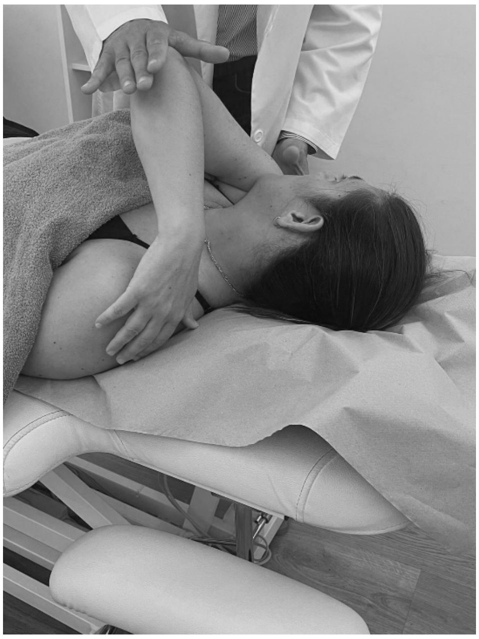

Figure S1. Physical therapist performing passive mobilisation in shoulder adduction.

Propioceptive neuromuscular facilitation

Movements	Series with each shoulder	Repetitions with each shoulder	Contraction time in seconds	Rest in seconds
**Kabat 1 diagonal: flexion, adduction and external rotation – extension, abduction and internal rotation.**	2	10	5 of isometric contraction -30 of passive stretching	30
**Kabat 2 diagonal: flexion, abduction and external rotation - extension, adduction and internal rotation**	2	10	5 of isometric contraction - 30 of passive stretching	30

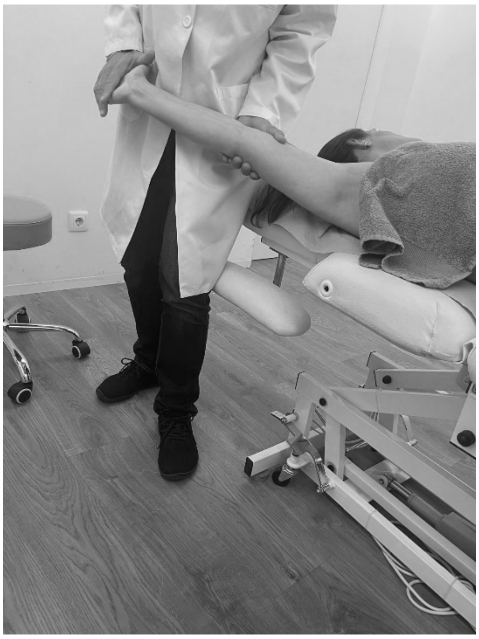

Figure S2. Physical therapist performing Kabat's diagonal 2.

Manual therapy

Technique	Duration on each shoulder
**Deep transverse massage (Cyriax technique): applied to the rotator cuff, peri-scapular area, and cervical region.**	10 minutes

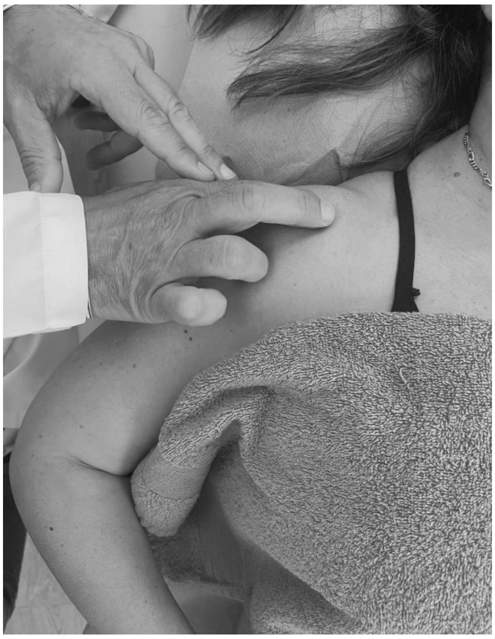

Figure S3. Physical therapist performing Cyriax massage

Passive stretching

Muscle	Series with each shoulder	Repetitions with each shoulder	Duration in seconds	Rest in seconds
**Trapezius: rotate and flex the patient's head toward the opposite shoulder and lower the shoulder of the trapezius muscle to be stretched.**	2	3	30	30
**Latissimus dorsi: full shoulder flexion and lateralisation of the trunk to the opposite side of the stretch.**	2	3	30	30
**Subscapularis: with the shoulder in a neutral position and the elbow at 90°, force external rotation.**	2	3	30	30
**External rotators: with the shoulder in a neutral position, force internal rotation.**	2	3	30	30
**Pectoralis: with a 90° shoulder abduction, bring the patient's arm backward.**	2	3	30	30

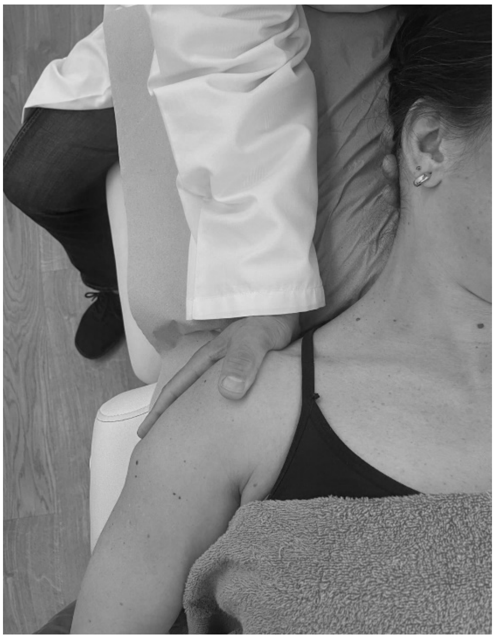

Figure S4. Physical therapist performing trapezius stretching.
